# Robust Visual Tracking Based on Adaptive Convolutional Features and Offline Siamese Tracker

**DOI:** 10.3390/s18072359

**Published:** 2018-07-20

**Authors:** Ximing Zhang, Mingang Wang

**Affiliations:** Academy of Astronautics, Northwestern Polytechnical University, YouYi Street, Xi’an 710072, China; mingangw@nwpu.edu.cn

**Keywords:** spatially regularized discriminative correlation filter (SRDCF)-based visual tracking, deep convolutional features, adaptive dimensionality reduction, adaptive model update, offline Siamese tracker

## Abstract

Robust and accurate visual tracking is one of the most challenging computer vision problems. Due to the inherent lack of training data, a robust approach for constructing a target appearance model is crucial. The existing spatially regularized discriminative correlation filter (SRDCF) method learns partial-target information or background information when experiencing rotation, out of view, and heavy occlusion. In order to reduce the computational complexity by creating a novel method to enhance tracking ability, we first introduce an adaptive dimensionality reduction technique to extract the features from the image, based on pre-trained VGG-Net. We then propose an adaptive model update to assign weights during an update procedure depending on the peak-to-sidelobe ratio. Finally, we combine the online SRDCF-based tracker with the offline Siamese tracker to accomplish long term tracking. Experimental results demonstrate that the proposed tracker has satisfactory performance in a wide range of challenging tracking scenarios.

## 1. Introduction

Target tracking is a classical computer vision problem with many applications. In generic tracking, the goal is to estimate the trajectory and size of a target in an image sequence, given only its initial information [1]. Target tracking has significantly progressed, but challenges still remain due to appearance change, scale change, deformation, and occlusion. Researchers have been tackling these problems by using the learning discriminative appearance model of the target. This method describes the target and background appearance based on rich feature representation. As such, this paper investigates deep robust feature representations, adaptive model updates, and Siamese offline tracker for robust visual tracking.

Danelljan et al. [2] proposed the spatial regularization correlation filter (SRDCF), which introduced learning to the penalize correlation filter coefficients depending on their spatial location. The SRDCF framework has been significantly improved by including scale estimation [3], non-linear kernels [4], long-term memory [5], and by removing the periodic effects of circular convolution [2,6,7]. However, three main problems limit the SRDCF formulation. Firstly, the dimension of the deep features significantly limits the tracking speed. Secondly, short-term target tracking algorithms cannot handle the out-of-view problem. Thirdly, online updates with fixed rate cause drift when suffering heavy occlusion.

Advances in visual tracking have been made for the features learned from deep convolutional neural networks (DCNNs). However, the outperforming deep features rely heavily on training on large-scale datasets. Thus, most state-of-the-art trackers use pre-trained networks to extract deep features. However, these improvements in robustness cause significant reductions in tracking speed. Siamese Networks have also been used to solve the tracking problem. The matching mechanism in Siamese Network approaches prevent model contamination and achieve better tracking performance. To perform long-term tracking, some methods implement a failure detection mechanism to combine multiple detectors with complementary characteristics at the different tracking stages. However, these approaches only use online update tracking and cannot unite the Siamese Trackers.

Based on the discussion above, we propose a novel SRDCF tracking framework that synthetically uses DCNN and failure detection combined with Siamese trackers. The main contributions of this paper are as follows: (1) We propose a method to obtain a specific feature map considering the tradeoffs between spatial information and semantic information though convolutional feature response, and use an adaptive projection matrix to obtain the principal component of the corresponding feature map, which reduces the computational complexity during feature extraction. (2) We propose a novel adaptive model updating method. First, we obtain the confidence of the target position based on the peak-to-sidelobe ratio (PSR), and then explore the confidence map to obtain the PSR, which is highly reliable. Finally, the weight is calculated by the given PSR and is used to achieve adaptive model updating. (3) We also combine the SRDCF frameworks with a Siamese Tracker by assigning the threshold; we infer the tracker status and warn of potential tracking failures in order to achieve long-term tracking by switching two different trackers.

The rest of the paper is organized as follows: in Section 2, we review related research work. In Section 3, we present the proposed visual tracking framework in detail. Numerous experimental results and analyses are shown in Section 4. In Section 5, we provide the conclusions to our work.

## 2. Related Work

### 2.1. Tracker with Correlation Filter

Discriminative Correlation Filters (DCFs) [2,8,9] have outstanding results for visual tracking. This approach uses the circular correlation properties to train a regressor using a sliding window. At first, DCF methods [8,10] were limited to a single feature channel. Some approaches have extended the DCF framework to multi-channel feature maps [11,12,13]. The high-dimensional features are exploited in multi-channel DCF for improved tracking. The combination of the DCF framework and deep convolutional features [14] has significantly improved tracking ability. Danelljan et al. [3] proposed scale estimation to achieve spatial evaluation. Danelljan et al. [2] also introduced spatial regularization in order to alleviate the boundary effect in SRDCF. Valmadre et al. [15] constructed a convolutional neural network (CNN) that contains a correlation filter as the part of the network and uses end-to-end representation learning based on the similarity between correlation and convolution operations.

### 2.2. Tracker with Deep Features

The introduction of CNNs has significantly progressed the field of computer vision, including visual tracking. Wang et al. [9] proposed a deep learning tracker (DLT) that is based on the combination of offline pre-training and online fine-tuning. Wang et al. [16] designed the structured output deep learning tracker (SO-DLT) within the particle filters framework. Trackers were introduced that learn target-specific CNNs without pre-training to prevent the problems caused by offline training, which treat the CNN as black box [17,18]. In order to learn multiple correlation filters, Ma et al. [19] extracted the hierarchical convolutional features (HCF) from three layers of related networks. Danelljan et al. [20] proposed a tracker by learning continuous convolution operators (CCOT) to interpolate discrete features and train spatial continuous convolution filters, which enabled the efficient integration of multi-resolution deep feature maps. Danelljan et al. [21] also designed an efficient convolution operator (ECO) for visual tracking using a factorized convolution operation to prevent the low computational efficiency caused by CNN operation. 

### 2.3. Trackers with Feature Dimensionality Reduction

Dimensionality reduction is widely used in visual tracking due to the computational complexity. Danelljan et al. [22] minimized the data term used in Principal Component Analysis (PCA) on the target appearance. In order to achieve sparse representation of the related target, Huang et al. [23] used sparse multi-manifold learning to achieve semi-supervised dimensionality reduction. Cai et al. [24] designed an adaptive dimensionality reduction method to handle the high-dimensional features extracted by deep convolutional networks. To model the mapping from high-dimensional SPD manifold to the low-dimensional manifold with an orthonormal projection, Harandi et al. [25] proposed a dimensionality reduction method to handle high-dimensional SPD matrices by constructing a lower-dimensional SPD manifold.

### 2.4. Trackesr with Siamese Networks

Siamese architecture has been exploited in the tracking field, performing impressively without any model update. Tao et al. [26] trained a Siamese network to identify candidate image locations that match the initial object appearance, and called their method the Siamese Instance Search Tracker (SINT). In this approach, many candidate patches are passed through the network, and the patch with the highest matching score is selected as the tracking output. Held et al. [27] introduced GOTURN, which avoids the need to score many candidate patches and runs at 100 fps. However, a disadvantage of their approach is that it does not possess intrinsic invariance for translating the search image. Later, Bertinetto et al. [28] trained a similar Siamese network to locate an example image within a large search image. The network parameters were initialized by the pre-trained networks through ILSVRC2012 (Large Scale Visual Recognition Challenge) [29] image classification problem, and then fine-tuned for the similarity learning problem in the second offline phase.

## 3. Proposed Method

### 3.1. Baseline

The SRDCF tracker [2] is a spatially regularized correlation filter obtained by exploiting the sparse nature of the proposed regularization in the Fourier domain. The tracker effectively reduces the boundary effect and has achieved better tracking performance in OTB2015 benchmark compared with other correlation filter tracking algorithms.

In the learning stage, the SRDCF tracker introduces a spatial weight function ω to penalize the magnitude of the filter coefficient f. The regularization weights ω determine the importance of the correlation filter coefficients f depending on their spatial locations. Coefficients in f residing outside the target region are suppressed by assigning higher weights to ω and vice versa. The resulting optimization problem is expressed as:

(1) $$\varepsilon \left(f\right)=\sum_{k=1}^{t}\alpha_{k}{\left\|S_{f}\left(x_{k}\right)-y_{k}\right\|}^{2}+\sum_{l=1}^{d}{\left\|\omega \cdot f^{l}\right\|}^{2}$$

where $\alpha_{k}\geq 0$ denotes the influence of sample $x_{k}$ to coefficient $f^{l}$. $S_{f}\left(x_{k}\right)=\sum_{l=1}^{d}x_{k}^{l}⋆f^{l}$ represents the convolution response of the filter to samples *x_k_* and *l* is the dimension of feature. The desired output *y_k_* is a scalar valued function over the domain that includes a label for each location in the sample, k denotes the number of frames, t represents the total number of samples, and d donates the dimension of the feature map.

By applying Parseval’s theorem to Equation (1), the filter f can equivalently be obtained by minimizing the resulting loss function in Equation (2) over the DFT coefficients $\hat{f}$:

(2) $$\overset{⌣}{\varepsilon }\left(\hat{f}\right)=\sum_{k=1}^{t}\alpha_{k}{\left\|\sum_{l=1}^{d}D\left({\hat{x}}_{k}^{l}\right){\hat{f}}^{l}-{\hat{y}}_{k}\right\|}^{2}+\sum_{l=1}^{d}{\left\|\frac{C\left(\hat{w}\right)}{MN}{\hat{f}}^{l}\right\|}^{2}$$

The symbol ${}^{⌢}$ denotes DFT, M,N represents the sample size, $D\left({\hat{x}}_{k}^{l}\right)$ denotes the diagonal matrix with the elements of the vector ${\hat{x}}_{k}^{l}$ in the diagonal, $C\left(\hat{w}\right)$ represents the circular two-dimensional (2D) convolution in the function (i.e.; $C\left(\hat{w}\right){\hat{f}}^{l}=vec\left(\hat{w}⋆{\hat{f}}^{l}\right)$), and vec(⋅) is the vector representation.

By applying unitary *MN* *×* *MN* matrix, B, and the real-valued part of ${\hat{f}}^{l}$, we obtain ${\tilde{f}}^{l}=B{\hat{f}}^{l}$. The loss function is then simplified by defining the fully vectorized real-valued filter as the concatenation $\tilde{f}={\left[{\left({\tilde{f}}^{1}\right)}^{T},\ldots ,{\left({\tilde{f}}^{d}\right)}^{T}\right]}^{T}$:

(3) $$\tilde{\varepsilon }\left(\tilde{f}\right)=\sum_{k=1}^{t}\alpha_{k}{\left\|D_{k}\tilde{f}-{\tilde{y}}_{k}\right\|}^{2}+{\left\|W{\hat{f}}^{l}\right\|}^{2}$$

where $D^{l}=\left(D_{k}^{1},\ldots ,D_{k}^{d}\right)$, $D_{k}^{l}=BD\left({\hat{x}}_{k}^{l}\right)B^{H}$, and ${\tilde{y}}_{k}=B{\tilde{y}}_{k}$, $C=BC\left(\hat{w}\right)B^{H}/MN$. We defined W as the dMN×dMN block diagonal matrix with each diagonal block being equal to C.

Finally, the regularized correlation filter is obtained by solving the normal equation $A_{t}\tilde{f}={\tilde{b}}_{t}$, where:

(4) $$A_{t}=\sum_{k=1}^{t}\alpha_{k}D_{k}^{T}D_{k}+W^{T}W$$

(5) $${\tilde{b}}_{t}=\sum_{k=1}^{t}\alpha_{k}D_{k}^{T}{\tilde{y}}_{k}$$

The SRDCF model is updated first by extracting a new training sample $x_{t}$ centered at the target location. Here, t denotes the current frame number. We then update $A_{t}$ in Equation (4) and ${\tilde{b}}_{t}$ in Equation (5) with a learning rate γ≥0:

(6) $$A_{t}=\left(1-\gamma \right)A_{t-1}+\gamma \left(D_{t}^{T}D_{t}+W^{T}W\right)$$

(7) $${\tilde{b}}_{t}=\left(1-\gamma \right){\tilde{b}}_{t-1}+\gamma D_{t}^{T}{\tilde{y}}_{t}$$

### 3.2. Adaptive Convolutional Features

**Figure 1:**
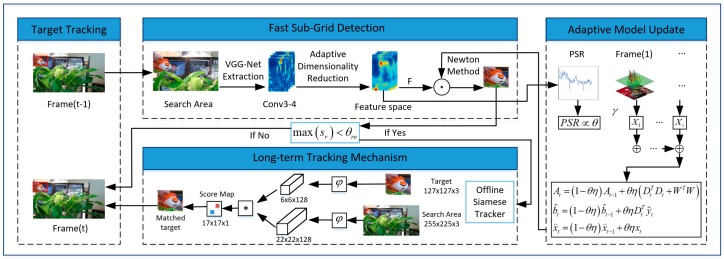
Flow chart of the proposed tracking algorithm.

By applying the convolutional features of the pre-trained VGG-Net [12], we used an adaptive dimension reduction method to construct the feature space, then designed the peak-to-sidelobe ratio to choose more reliable results in order to update the model. For long-term tracking, we designed a novel failure detection mechanism in the tracking procedure. By combining the online updating method and the offline tracker, we not only addressed the issues in the SRDCF framework, but also solved the occlusion, deformation, and out-of-view problems present in long-term tracking. The flow chart of proposed the tracking algorithm is shown in Figure 1.

#### 3.2.1. Convolutional Features

Convolutional neural networks (CNNs) have successfully applied to large image classification and detection by extracting features or by directly performing the task, such as with AlexNet [30], GoogleNet [31], ResNet [32], and VGG-Net [12]. VGG-Net was trained by 1.3 million images in the ImageNet dataset, and achieved the best result in a classification challenge. Compared with most CNN models of only five to seven layers, VGG Net has a deeper structure with up to 19 layers, 16 convolution and three fully-connected layers, which contain spatial information and semantic information, respectively, which can identify deeper features. 

**Figure 2:**
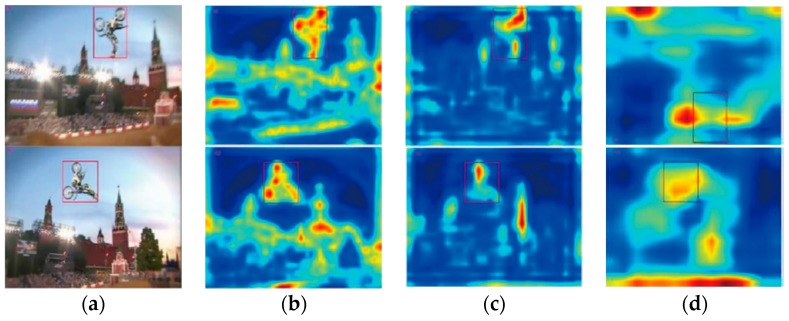
Convolutional features response: (**a**) original images, (**b**) Conv3-4, (**c)** Conv4-4, and (**d**) Conv5-4.

Research indicates that the features extracted by convolution layer features are better than extracted from fully-connected layers. As shown in Figure 2, the feature extracted by the Conv3-4 layer in the VGG-Net model maintains spatial details, especially some information that is useful for accurate tracking (Figure 2b). Figure 2d illustrates the Conv5-4 layer of the VGG-Net model, which contains more semantic information. The semantic information effectively achieves better feature extraction when experiencing deformation in the tracking process. We chose the Conv3-4 feature in this paper considering the tradeoff between spatial information and semantic information.

The feature mapping of Pool5 is only 7 × 7. Achieving accurate location depending on such low resolution is impossible. Bilinear interpolation is typically used to solve this problem in mapping space,

(8) $$x_{k}=\sum_{i}\beta_{ki}h_{i}$$

where the weight $\beta_{ki}$ depends on the location of *k*th frame and *i*th adjacent eigenvectors, and **h** represents the feature space.

#### 3.2.2. Adaptive Dimensionality Reduction

**Figure 3:**
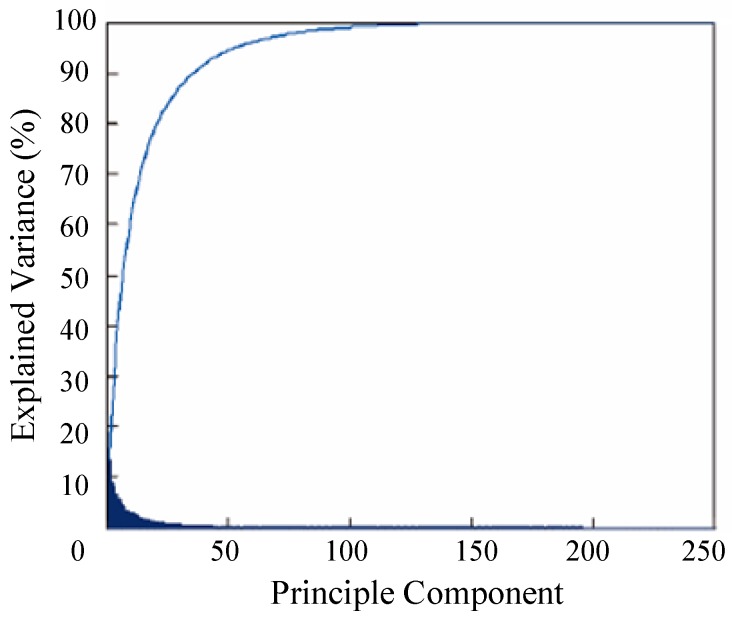
Principal component analysis of features from Conv3-4 on MotorRolling.

The feature dimension of Conv3-4 layer is 56 × 56 × 256, which contains less information and increases computation time. We used an adaptive dimensionality reduction to preserve the main component of Conv3-4, depending on the principal component analysis (PCA) of the related layer. After applying this method, the feature dimension was reduced to 130 from 256. As shown in Figure 3, the contribution of the feature under adaptive dimensionality reduction was 98% in sequence MotorRolling.

${\hat{x}}_{t}$ denotes the *D*_1_-dimensional feature learned from frame *t*. Adaptive dimensionality reduction results in the projection matrix $P_{t}$, which contains an orthogonal vector in $D_{1}\times D_{2}$ dimension, and $P_{t}^{T}P_{t}=I$. By applying the projection matrix $P_{t}$, we achieved the new *D*_2_-dimensional feature space:

(9) $$\min\left\{\eta_{t}\left[\frac{1}{MN}\sum_{m,n}{\left\|{\hat{x}}_{t}\left(m,n\right)-P_{t}P_{t}^{T}{\hat{x}}_{t}\left(m,n\right)\right\|}^{2}\right]+\sum_{k=1}^{t-1}\left[{\sum_{l-1}^{D_{2}}\xi_{k}^{\left(l\right)}\left\|r_{k}^{\left(l\right)}-P_{t}P_{t}^{T}r_{k}^{\left(l\right)}\right\|}^{2}\right]\right\}$$

where $\eta_{1},\ldots ,\eta_{t}$ denote weights and $\xi_{k}^{\left(l\right)}\geq 0$ determines the importance of each component vector $r_{k}^{\left(l\right)}$, where ${\hat{x}}_{t}\left(m,n\right)=P_{t}^{T}{\hat{x}}_{t}\left(m,n\right),\forall m,n$.

We used singular value decomposition (SVD) of the matrix $R_{t}$ to solve Equation (9). The projection matrix is chosen from the first $D_{2}$ feature vectors from matrix $R_{t}$:

(10) $$R_{t}=\eta_{t}G_{t}+\sum_{k=1}^{t-1}\eta_{k}P_{k}\Lambda_{k}P_{k}^{T}$$

(11) $$G_{t}=\frac{1}{MN}\sum_{m,n}\left\|{\hat{x}}_{t}\left(m,n\right)-{\bar{x}}_{t}\right\|{\left\|{\hat{x}}_{t}\left(m,n\right)-{\bar{x}}_{t}\right\|}^{T}$$

(12) $${\bar{x}}_{t}=\frac{1}{MN}\sum_{m,n}{\hat{x}}_{t}\left(m,n\right)$$

where $G_{t}$ denotes the covariance matrix of; $\Lambda_{t}$ represents the diagonal matrix with $D_{2}\times D_{2}$, which contains $\xi_{k}^{\left(l\right)}$ in the diagonal position; and $\xi_{k}^{\left(l\right)}$ denotes the eigenvalue of component vector $r_{k}^{\left(l\right)}$ corresponding to the matrix $R_{t}$.

We obtain the adaptive projection matrix though a fixed learning rate λ. The matrix $R_{t}$ and the variance matrix $Q_{t}$ are updated using linear interpolation at every time step. Use the fixed learning rate γ≥0 to simultaneously update the appearance feature space ${\hat{x}}_{t}$. $x_{t}$ donates the feature space determined through Equation (8). Due to the Pooling operation, the feature space contains more semantic information:

(13) $$Q_{t}=\left(1-\lambda \right)Q_{t-1}+\lambda P_{t}\Lambda_{t}P_{t}^{T}$$

(14) $$R_{t}=\left(1-\lambda \right)Q_{t-1}+\lambda G_{t}$$

(15) $${\hat{x}}_{t}=\left(1-\gamma \right){\hat{x}}_{t-1}+\gamma x_{t}$$

#### 3.2.3. Fast Sub-Grid Detection

At the detection stage, the location of the target in a new frame t is estimated by applying the filter ${\hat{f}}_{t-1}$ that was updated in the previous frame. Apply the filter at multiple resolutions to estimate changes in the target size. The samples ${\left\{z_{r}\right\}}_{r\in \left\{\left[\left(1-S\right)/2\right],\ldots ,\left[\left(S-1\right)/2\right]\right\}}$ are extracted, centered at the previous target location and at the scale $a^{r}$ relative to the current target scale. Here, S denotes the number of scales and a is the scale increment factor. The sample $z_{r}$ is constructed by resizing the image according to $a^{r}$ before feature computation.

Use an interpolation approach that allows computation of pixel-dense detection scores. The detection scores are efficiently interpolated with trigonometric polynomials by using the computed DFT coefficients. Let $\hat{s}:=F\left\{S_{f}\left(z\right)\right\}=\sum_{l=1}^{d}{\hat{z}}^{l}$, and ${\hat{f}}^{l}$ be the DFT of the scores $S_{f}\left(z\right)$ evaluated at sample z. The detection scores $s_{r}\left(u,v\right)$ at the continuous location (u,v)∈[0,M)×[0,N) in z are interpolated as:

(16) $$s_{r}\left(u,v\right)=\frac{1}{MN}\sum_{0}^{M-1}\sum_{0}^{N-1}{\hat{s}}_{r}\left(m,n\right)\exp\left[i2\pi \left(\frac{m}{M}u+\frac{n}{N}v\right)\right]$$

where i denotes the imaginary unit. We iteratively maximize Equation (16) using Newton’s method by starting at the location $\left(u^{\left(0\right)},v^{\left(0\right)}\right)\in \Omega$. The gradient and Hessian in each iteration are computed by analytically differentiating Equation (16) to the maximum score:

(17) $$\left(u^{*},v^{*},r^{*}\right){=\mathrm{argmax}}_{\left(u,v\right)\in \left[0,M\right)\times \left[0,N\right)}s_{r}\left(u,v\right)$$

#### 3.2.4. Adaptive Model Update

The SRDCF framework uses the fixed learning rate to update the tracking model. Once the target is occluded, the appearance model is negatively affected, which leads to tracking drift. The proposed method uses the PSR $R_{PSR}$ to compute the confidence of the target position [33]. Through this method, we update the model depending on the confidence. PSR has been widely used in signal processing; usually the peak intensity of the signal can be expressed as:

(18) $$R_{PSR,t}=\frac{\max\left[S_{f}\left(x_{t}\right)\right]-\varphi_{t}}{\sigma_{t}}$$

where $S_{f}\left(x_{t}\right)$ represents the convolution response to the correlation filter of the sample, and $\varphi_{t}$ and $\sigma_{t}$ denote the mean and standard deviation of convolution response to the sample $x_{t}$, respectively.

**Figure 4:**
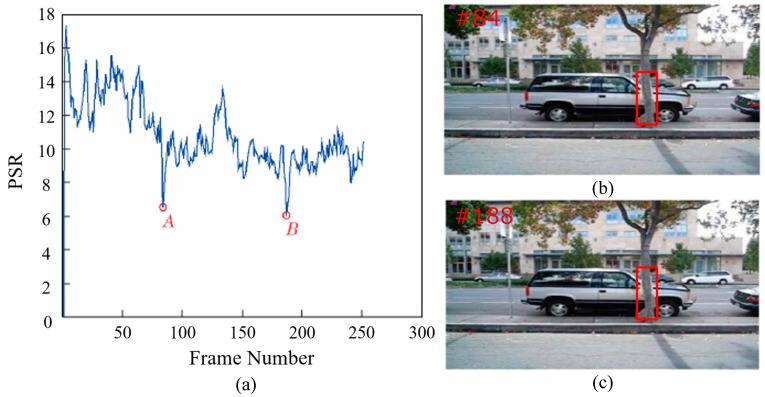
Analysis and (**a**) distribution of the peak-to-sidelobe ratio (PSR) on the David3 dataset: (**b**) 84th and (**c**) 188th frame of the David3 dataset.

The PSR distribution of the David3 dataset is shown in Figure 4. The higher the PSR, the higher the confidence score of the target location. The target is completely occluded by the tree in the 84th frame, so the corresponding PSR drops to the extreme point, as seen in point A in Figure 4. The PSR gradually increase in the following frames. When the target was completely occluded by the trees in the 188th frame, the corresponding PSR decreases to the extreme point again, as shown by point B in Figure 4. The tracking results of point A and B are apparently unreliable, which cannot be used to update the model. The experiments show that the tracking result is highly reliable when PSR is around 10–18. 

Therefore, it is possible to determine whether the target is affected by the occlusion according to PSR in order to assign weight to the model update:

(19) $$\theta =\{\begin{array}{cc} 0.1R_{PSR} & \mathrm{if}\;R_{PSR}\geq 10\\ 0 & \mathrm{if}\;R_{PSR}<10 \end{array}$$

The model is updated by using the learning rate η as follows:

(20) $$A_{t}=\left(1-\theta \eta \right)A_{t-1}+\theta \eta \left(D_{t}^{T}D_{t}+W^{T}W\right)$$

(21) $${\tilde{b}}_{t}=\left(1-\theta \eta \right){\tilde{b}}_{t-1}+\theta \eta D_{t}^{T}{\tilde{y}}_{t}$$

(22) $${\hat{x}}_{t}=\left(1-\theta \eta \right){\hat{x}}_{t-1}+\theta \eta x_{t}$$

### 3.3. Long-Term Tracking Mechanism Based on Siamese Offline Tracker

Studies have shown the impressive performance of Siamese networks without any model update [26,27,28]. Compared with online trackers, these Siamese-network-based offline trackers are more robust to noisy model updates. Moreover, state-of-the-art tracking performance was achieved with a rich representation model learned from the large IILSVRC15 dataset [29]. However, these Siamese-network-based offline trackers are prone to drift in the presence of distractors that are similar to the target or when the target appearance in the first frame is significantly different from that in the remaining frames. Motivated by the complementary traits of online and offline trackers, we equipped our online update method with an offline-trained fully convolutional Siamese network [28]. By using this method, the stability-plasticity dilemma was balanced. 

In long term tracking, tracking-learning-detection (TLD) [34] implements the long-term tracking mechanism in each frame of the image sequence. The proposed algorithm used threshold $\theta_{re}$ to activate the long-term tracking mechanism. When $\max\left(s_{r}\right)<\theta_{re}$, the tracking method switches to the offline Siamese tracker. When $\max\left(s_{r}\right)$ is less than the activation threshold, the algorithm elects the offline Siamese tracker to track the target. The process is executed once, when $\max\left(s_{r}\right)<\theta_{re}$. The implementation details of the fully convolutional Siamese Network were provided in a previous study [28]. The ablation study in Section 4.2 shows that the proposed offline tracker can avoid noisy model updates to achieve some improvements. The overall tracking algorithm is described in Algorithm 1. 

**Algorithm 1:** Proposed tracking algorithm.**Input:** Image I; Initial target position $\left(u_{\left(0\right)},v_{\left(0\right)}\right)$ and scale $a^{r_{0}}$; previous target position $\left(u_{\left(t-1\right)},v_{\left(t-1\right)}\right)$ and scale $a^{r_{t-1}}$
**Output:** Estimated object position $\left(u_{\left(t\right)},v_{\left(t\right)}\right)$ and scale $a^{r_{t}}$.
**For each**
$I_{t}$
 Extract the deep feature space $\hat{x_{t}}$ thought the pre-trained VGG-Net; Update matrix $R_{t}$ and $Q_{t}$ by linear interpolation using Equation (13) and (14). The SVD is performed and a new $P_{t}$ is found; Update the low dimensional appearance feature space ${\hat{x}}_{t}$ using Equation (15); Compute the confidence of the target position using Equation (18); Update the tracking model $A_{t}$, ${\tilde{b}}_{t}$ and ${\hat{x}}_{t}$ using Equations (19)–(22); Compute the estimated object position $\left(u_{\left(t\right)},v_{\left(t\right)}\right)$ and scale $a^{r_{t}}$ using fast sub-grid detection; If $\max\left(s_{r}\right)<\theta_{re}$, Update the estimated object position and scale using the offline Siamese tracker; Else Output the estimated object position and scale directly;
**End**


## 4. Experimental Results and Analysis

This section presents a comprehensive experimental evaluation of the proposed tracker. 

### 4.1. Implementation Details

The configuration used was an Intel (R) Core ™ I74790 CPU, 3.6 GHz, 16 GB RAM, NVIDIA Tesla K20 m GPU standard desktop. The weight function ω was constructed by starting from a quadratic function $\omega \left(m,n\right)=\tau +\xi \left\{{\left(m/P\right)}^{2}+{\left(n/Q\right)}^{2}\right\}$. The minimum value of ω was set to ω=τ=0.1, and the impact of the regularizer was set to ζ=3. P×Q denotes the target size. The number of the scale was set to S=7, and a=1.01 denotes the scale incremental factor. During adaptive dimensionality reduction, the feature dimension of Conv3-4 was set to $D_{1}=256$, which was reduced to $D_{2}=130$. During linear interpolation, the learning ratio was set to λ=0.15, γ=0.025. $\theta_{re}=0.5$ was used to activate the offline Siamese tracker; the tracker used the same parameters as in a previous study [20]. The $R_{PSR,t}$ was set to 10 during model update, and the learning ratio was set to η=0.01. Our MATLAB implementation ran at 4.6 frames per second with MatConvNet [35].

### 4.2. Reliablity Ablation Study

An ablation study on VOT2016 was conducted to evaluate the contribution of the adaptive dimensionality reduction, adaptive model update, and Siamese tracker in the proposed method. The results of the VOT primary measure expected average overlap (EAO) and two supplementary measures, accuracy (A) and robustness (R), are summarized in Table 1 We provide the details of performance measures and evaluation protocol of VOT2016 in Section 4.4. Performance of the various modifications of the proposed method are discussed in the following.

Applying the adaptive dimensionality reduction reliability is equivalent to extracting the principle component from the original image feature space. It not only reduces the computational complex, but also improves the sematic representation during the procedure. The performance drop in EAO compared to the proposed method was 11%.

Replacing the adaptive model updating means that $Ours_{Adr-}$ does not use the PSR ($R_{PSR}$) to compute the confidence of the target position and completed the updating procedure based on the confidence. Since the updated filter drifted due to the deformation and occlusion, which affect the appearance of the tracking object, this version reduced our tracker performance by over 22% in EAO. $R_{av}$ remained unchanged in this experiment, whereas the $A_{a}{}_{v}$ of this version dropped by over 40%.

Replacing the Siamese tracker from the proposed method mainly affected the performance of long-term tracking. The performance drop in EAO compared with the proposed method was around 10%, and the $A_{a}{}_{v}$ dropped 20% due to the lack of a failure detection mechanism. This clearly illustrates the importance of our combination of the online tracker and Siamese tracker as outlined in Section 3.3.

### 4.3. OTB-2015 Benchmark

**Figure 5:**
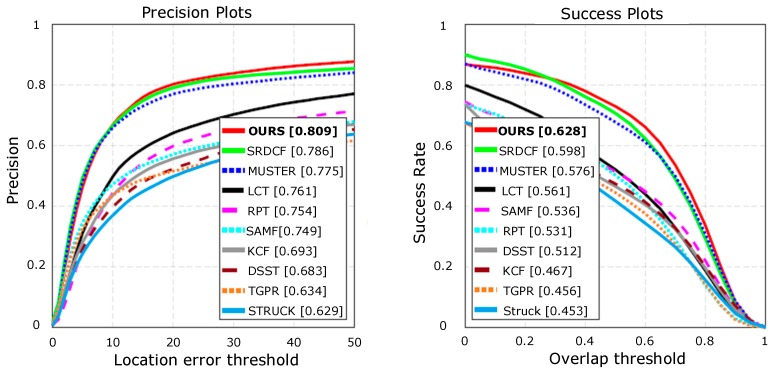
Evaluation on OTB100 benchmark.

The OTB100 [36] benchmark contains the results of 29 trackers evaluated on 100 sequences using a no-reset evaluation protocol. We measured the tracking quality using precision and success plots. The success plot shows the fraction of frames with an overlap between the predicted and ground truth bounding box greater than a threshold with respect to all threshold values. The precision plot shows similar statistics on the center error. The results are summarized by areas under the curve (AUC) in these plots. Here, we only show the results for top-performing recent baselines to avoid clutters, including Struck [8], TGPR [37], DSST [3], KCF [4], SAMF [38], RPT [39], LCT [5], and results for recent top performing state-of-the-art trackers SRDCF [2] and MUSTER [40]. The results are shown in Figure 5. The proposed method performed the best in OTB100 and outperformed the baseline tracker, SRDCF. The OTB success plots computed on these trajectories and summarized by the AUC values are equal to the average overlap [41].

### 4.4. VOT2016 Benchmark

We compared the proposed tracker with other state-of-the-art trackers in VOT2016, which contains 60 sequences. The trackers were restarted at each failure. The set is diverse, with the top-performing trackers come from various classes including correlation filter methods such as CCOT [20], ECO [21], Staple [42], and DDC [43]; deep convolutional network methods such as TCNN [43], SSAT [44], MLDF [45], and SiamFC [28]; and different detection-based approaches such as EBT [46] and SRBT [43].

The proposed method outperforms the compared trackers, except for ECO and CCOT, with an EAO score of 0.329. The proposed method significantly outperformed the correlation filter approaches that apply deep ConvNets, and also outperforms the trackers that apply different detection-based approaches. The detailed performance scores for the 10 top-performing trackers are shown in Table 2.

### 4.5. Per-Attribute Analysis

**Figure 6:**
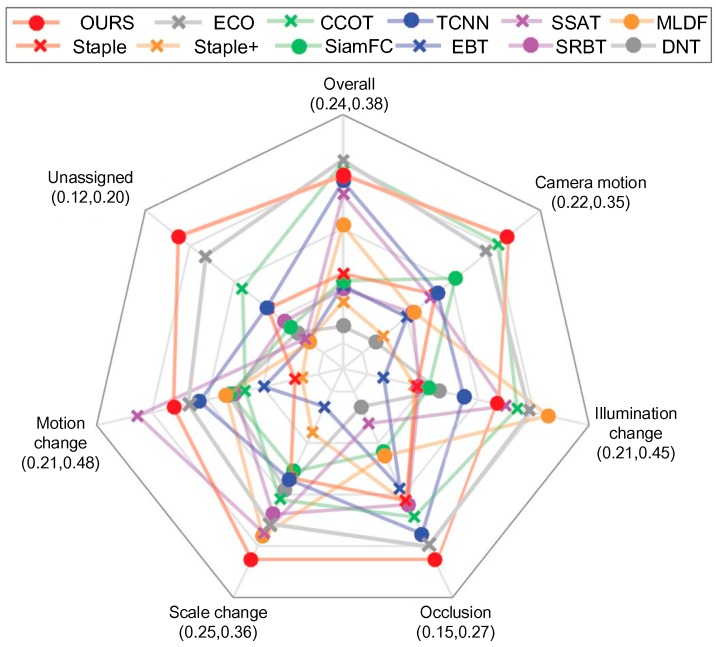
Expected averaged overlap performance on different visual attributes on the VOT2016 benchmark. The proposed method and the top performing trackers from VOT2106 are shown. The visual attribute axes are shown below the attribute labels.

The VOT2016 dataset is per-frame annotated with visual attributes to allow the detailed analysis of per-attribute tracking performance. Figure 6 shows the per-attribute plot for the top-performing trackers on VOT2016 in EAO. The proposed method was consistently ranked among the top three trackers on the five attributes. The proposed method performed the best in terms of size change, occlusion, camera motion, and unassigned. During the illumination change challenge, the proposed tracker did not perform better than four trackers, including ECO, CCOT, MLDF, and SSAT. 

### 4.6. Tracking Speed Analysis

Speed measurements on a single CPU were computed using an Intel^®^ Core™ I74790 CPU, 3.6 GHz, 16 GB RAM, NVIDIA Tesla K20 m GPU standard desktop. Compared with the two best-performing methods, ECO and CCOT, the proposed method was slower than ECO, while being four times faster than CCOT. Compared with other trackers that apply deep ConvNets, such as DeepSRDCF [14] and SiamFC, the proposed tracker had better tracking results and was twice as fast as DeepSRDCF. The proposed tracker performs nearly two times slower than the baseline SRDCF, but achieved better tracking results. Compared with baseline real-time trackers like KCF, DSST, and Staple, the proposed tracker performed poorly, but the tracking performance of the proposed tracker was much better. The speed of trackers in terms of frames per second is shown in Table 3.

**Figure 7:**
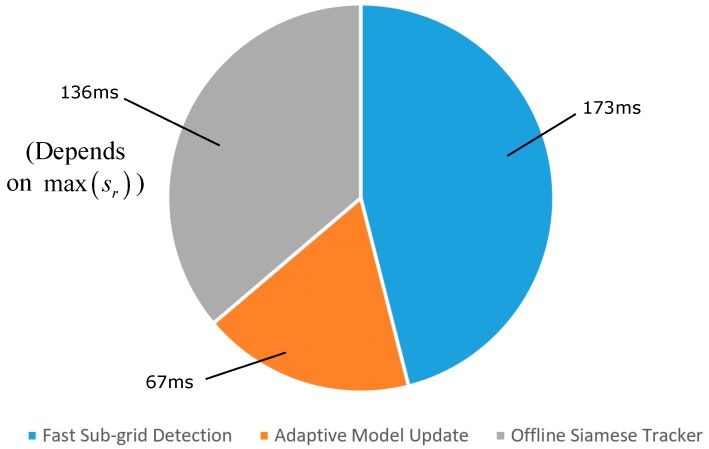
A single iteration processing time decomposed across the main steps of the proposed method.

The average speed of the proposed tracker measured on the VOT 2016 dataset was approximately 4.6 fps or 217 ms/frame. Figure 7 shows the processing time required by each step of the proposed method. Among them, the Fast Sub-Grid Detection process required 173 ms, the Adaptive Model Update required 67 ms, and the offline Siamese Tracker required 136 ms. The condition $\max\left(s_{r}\right)$ depends on whether or not the offline Siamese Tracker is employed. Due to the adaptive dimensionality reduction, the proposed tracker can save time than when directly using deep features.

### 4.7. Qualitative Evaluation

#### 4.7.1. Qualitative Evaluation on the OTB Benchmark

**Figure 8:**
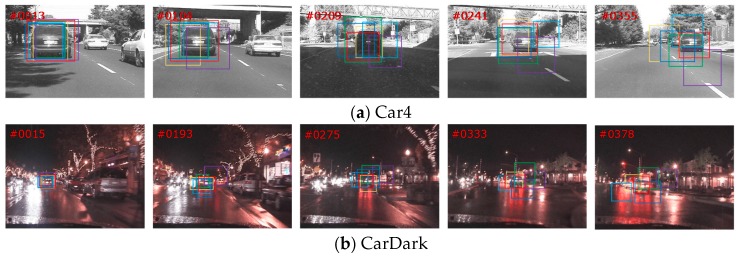

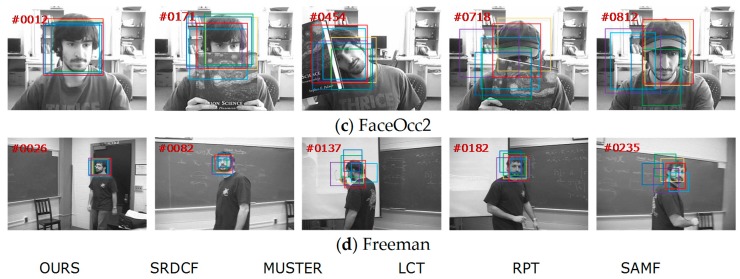
Tracking results of object undergoing severe occlusion, illumination, and in-plane rotation on OTB100. From top to bottom, the name of the video is (**a**) Car4; (**b**) CarDark; (**c**) FaceOcc2; (**d**) Freeman.

In this section, we focus on the tracking results for objects experiencing severe occlusion, illumination, and in-plane rotation on OTB100. The compared trackers included the baseline SRPDCF, MUSTER, LCT, RPT, and SAMF. The tracking results are shown in Figure 8. Given the rich representation of deep ConvNet, the proposed tracker outperformed other trackers given complex attributes. In sequence Car4 and CarDark, the illumination occurs in frames 205 and 333, respectively. In the sequence FaceOcc2, the target is occluded by a cap and book. In the Freeman sequence, the target is suffering from severe in-plane rotation. Due to the adaptive model update, the model is updated based on the peak-to-sidelobe ratio, which prevents the correlation filter from learning background information and tracking the object. Due to the deep ConvNet features, the proposed tracker contains rich representation that performs well when experiencing illumination change in the Car 4 and CarDark sequences. Notably, the proposed tracker succeeds in tracking the target until the very end of the FaceOcc2 and Freeman sequences. The offline Siamese Tracker is activated to achieve long-term tracking to prevent tracking failure from the online model update.

#### 4.7.2. Qualitative Evaluation on VOT Benchmark

**Figure 9:**
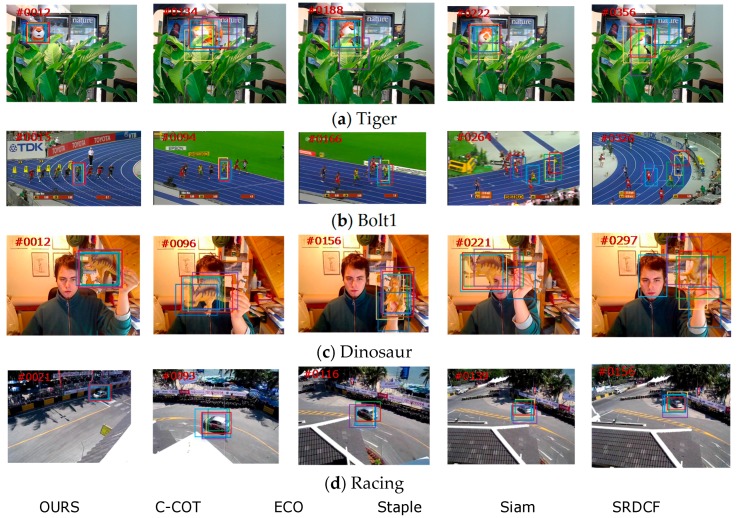
Tracking results of objects undergoing severe occlusion, scale change, and camera change on VOT2016. From top to bottom, the name of the video is (**a**) Tiger; (**b**) Bolt1; (**c**) Dinosaur; (**d**) Racing.

In this section, we focus on the tracking results of objects undergoing severe occlusion, scale change, and camera motion on VOT2016. The compared trackers included CCOT, ECO, Staple, SiamFC, and the baseline SRDCF. The tracking results are shown in Figure 9. The proposed tracker outperformed the other trackers in terms of occlusion, scale change, and camera change, which is illustrated in Section 4.5. In the Tiger sequence, the target is occluded frequently during the entire procedure. The tracker based on deep ConvNet performed well in this sequence, since the high number of layers retains rich semantics information. In the Bolt1 and Dinosaur sequence, the target experiences scale change. Compared with the other trackers, the proposed tracker performed well, due to the long-term mechanism of the offline Siamese tracker. In the Racing sequence, the camera changes throughout the sequence. Nearly all the trackers can track the target successfully, whereas the proposed tracker achieved the most accurate tracking, which can be seen in Figure 9d.

## 5. Conclusions

In this paper, we propose a visual tracking framework that combines deep ConvNet features, adaptive model updates, and an offline Siamese tracker. The proposed tracker outperformed other state-of-the-art methods in complex attributes. The adaptive dimensionality reduction provides low dimensional features for the correlation filter to reduce computational complexity. The adaptive model updating method improves the tracking performance in occlusion situations. The offline Siamese tracker enables long-term tracking. Numerous experimental results demonstrated that the proposed tracker outperforms state-of-the-art trackers, highlighting the significant benefits of our method.

## Figures and Tables

**Table 1:** Ablation study of the proposed method. The use of adaptive dimensionality reduction is indicated in the Adr. column and the use of the adaptive model updating is in the Amu. column. The St. column indicates whether to employ Siamese tracker.

Tracker	Adr.	Amu.	St.	EAO	$A_{a}{}_{v}$	$R_{av}$
Ours	x	x	x	0.329	0.59	0.83
$Ours_{St-}$	x	x	-	0.293	0.49	1.12
$Ours_{Adr-}$	-	x	x	0.282	0.47	0.87
$Ours_{Amu-}$	x	-	x	0.256	0.48	1.32
$Ours_{baseline}$	-	-	-	0.228	0.45	1.58

**Table 2:** The outperforming trackers on the VOT2016 benchmark.

Tracker	EAO	$A_{a}{}_{v}$	$R_{av}$
Ours	0.329	0.59	0.83
ECO	0.374	0.54	0.76
CCOT	0.331	0.52	0.85
TCNN	0.325	0.54	0.96
SSAT	0.321	0.57	1.04
MLDF	0.311	0.48	0.83
Staple	0.295	0.54	1.35
DDC	0.293	0.53	1.23
EBT	0291	0.44	0.90
SiamFC	0.284	0.52	0.87
SRBT	0.286	0.55	1.32

**Table 3:** Speed of trackers related in frames per second (fps).

Tracker	OURS	CCOT	ECO	SiamFC	SRDCF	DeepSRDCF	Staple	DSST	KCF
Average Fps	4.6	1.2	6.6	8.1	7.3	2.8	62.3	17.4	112.4
